# A novel *KCND3* mutation associated with early-onset lone atrial fibrillation

**DOI:** 10.18632/oncotarget.23303

**Published:** 2017-12-14

**Authors:** Yuan Huang, Jiawei Yang, Wanyi Xie, Qince Li, Zhipeng Zeng, Haibo Sui, Zhonggui Shan, Zhengrong Huang

**Affiliations:** ^1^ National “111” Center for Cellular Regulation and Molecular Pharmaceutics, Key Laboratory of Fermentation Engineering, Ministry of Education, Hubei University of Technology, Wuhan 430068, China; ^2^ Department of Cardiology, Jingzhou Central Hospital, The Second Clinical Medical College, Yangtze University, Jingzhou 434020, China; ^3^ Xiamen Key Laboratory of Chiral Drugs, Medical College, Xiamen University, Xiamen 361003, China; ^4^ Biocomputing Research Center, School of Computer Science and Technology, Harbin Institute of Technology, Harbin 150001, China; ^5^ Department of Cardiology, The First Affiliated Hospital of Xiamen University, Xiamen 361003, China; ^6^ Department of Cardiovascular Surgery, The First Affiliated Hospital of Xiamen University, Xiamen 361003, China

**Keywords:** atrial fibrillation, *KCND3*, transient outward potassium current, patch clamp

## Abstract

Atrial fibrillation (AF) is the most common arrhythmia in the clinic. While previous studies have identified AF-associated mutations in several genes, the genetic basis for AF remains unclear. Here, we identified a novel T361S missense mutation in *potassium voltage-gated channel, shal-related subfamily, member 3* (*KCND3*) from a Chinese Han family ancestor with lone AF. The wild-type (WT) or mutant T361S of K_v_4.3 protein (encoded by *KCND3*) were co-expressed with the auxiliary subunit K^+^ channel-Interacting Protein (KChIP2) in HEK293 cells, and transient outward potassium current (*I*_to_) were recorded using patch-clamp methods, and the surface or total protein levels of K_v_4.3 were analyzed by western blot. *I*_to_ density, measured at 60 mV, for T361S was significantly higher than that for WT. Both the steady-state activation and inactivation curves showed a remarkable hyperpolarizing shift in T361S. Moreover, recovery from inactivation after a 500-ms depolarizing pulse was significantly delayed for T361S compared with that for WT. Mechanistically, the gain of function of *I*_to_ elicited by T361S was associated with the increased expression of cell surface and total cell protein of K_v_4.3. The computer stimulation revealed that the T361S mutation shortened the action potential duration through an increased *I*_to_in Human Atrial Model. In conclusion, we identified a novel T361S mutation in *KCND3* associated with AF in the Chinese Han family. The T361S mutant result in the changes in channel kinetics as well as the up-regulation of K_v_4.3 protein, which may be a critical driver for lone AF as observed in the patient.

## INTRODUCTION

Atrial fibrillation (AF) is the most common sustained cardiac arrhythmia that results in serious cardiovascular outcomes such as stroke, heart failure and death [1]. The prevalence of AF is approximately 1% in the general population and markedly increases in aging populations, occurring in approximately 10% of individuals over age 80. AF is predicted to affect more than 33 million individuals worldwide [2]. AF is associated with a five-fold increased risk in the incidence of stroke and other cardiovascular mortalities and is rapidly becoming a public health challenge worldwide [3].

However, the defined etiology and pathogenesis of AF are incompletely understood. AF is usually associated with recognizable overt cardiovascular disease or precipitating illness, but more than 30% AF cases occurs in the absence of these complications (conventionally referred to as lone AF) [4, 5]. Genetic susceptibility is associated with the development of AF, especially to lone AF. Following the identification of the S140G mutation in *KCNQ1* in familial AF, mutations have also been identified in genes encoding ion channels and their accessory subunits, including sodium channels (*SCN5A* and β subunits) and potassium channels [6–10]. Subsequent studies found that mutations in non-ion channel genes are also linked to AF, such as connexins, atrial natriuretic peptide gene *NPPA*, T-box transcription factor 5 (*TBX5*) and nuclear pore complex 155 (*NUP155*) [10–13]. However, given that the aforementioned gene mutations occur in low prevalence in AF patients, it is reasonable to hypothesize that additional disease genes remain to be identified.

The rapidly activating and inactivating transient outward potassium currents (*I*_to_), mediated by the pore-forming potassium channel subunit K_v_4.3 (encoded by the *KCND3* gene) together with the K^+^ channel-interacting protein 2 (KChIP2, encoded by the *KCNIP2* gene), plays a vital role in the early phase of repolarization and excitation-contraction coupling [14, 15]. Dysfunctions of *I*_to_ contribute to pathophysiological conditions, including cardiac hypoxia and arrhythmia. Previous studies revealed that hypoxia (e.g., myocardial infarction) markedly decreased the *I*_to_ densities by a Ca^2+^-dependent mechanism [16–18]. In addition, a mutation in *KCNE3* (V17M), an accessory subunit that interacts with the K_v_4.3, leads to increased *I*_to_ and has been reported to be associated with the pathogenesis of early-onset long AF [19]. Multiple mutations in *KCND3* have been reported to be associated with the pathogenesis of dominantly inherited spinocerebellar ataxia (SCA) 19/22, Brugada syndrome (BrS) and sudden unexplained death syndrome [20–22]. A mutation in *KCND3* has also been reported in the pathogenesis of AF in Danish cohort populations [23]. However, mutation screening of the *KCND3* gene obtained from patients of Chinese Han ancestry with early onset lone AF has not been reported.

In the present study, given the genetic susceptibility of different racial groups, we pursued the identification of novel lone AF-associated mutations among individuals of Chinese Han ancestry.

## RESULTS

### Study cohort and mutation screening

**Figure 1:**
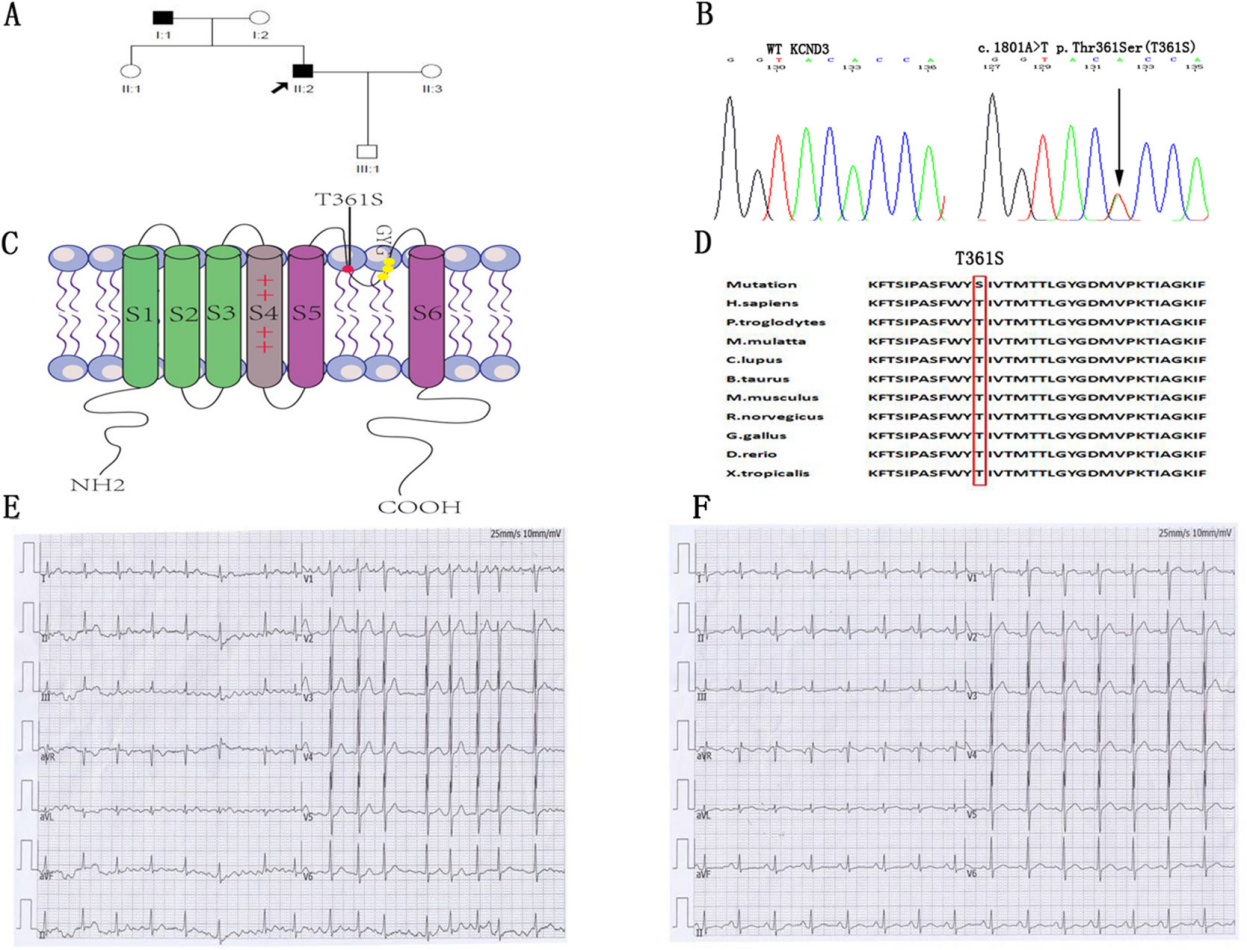
Identification of the mutant residues at position 361 in *KCND3* associated with lone AF **(A)** Family pedigree showing squares that indicate male family members, circles that indicate female family members and arrows that indicate the proband patient. His father was affected by persistent AF at age 38. **(B)** Direct sequencing chromatograms of the *KCND3* gene in the index patient and control case showing a heterozygous transition c.1081A>T resulting in replacement of threonine by serine at position 361. **(C)** Predicted topology schematic of K_v_4.3 protein showing the position of the T361S mutation in the third extracellular loop linking segment 5 and segment 6, which is regarded as channel pore-forming. **(D)** Amino acid sequence alignment showing that serine at position 361 is highly conserved among multiple species. **(E)** The proband patient had onset of paroxysmal AF at the age of 28. ECG data: HR 102 bpm; no P-wave; QRS 98 ms. **(F)** In sinus rhythm, the patient had a normal ECG (ECG data: HR 90 bpm; P-wave duration 98 ms; PR 168 ms; QRS 96 ms; QTc 392 ms).

A total of 180 patients with lone AF ranging from 16 to 39 years, without any concomitant disease, were screened for mutations in the cardiac potassium channel subunit K_v_4.3, encoded by *KCND3*. The clinical data from the study population are shown in Table 1. A heterozygous nucleotide variant of *KCND3* (c.1081A>T) leading to the substitution of a threonine residue at position 361 by a serine residue (label T361S) was identified in the proband AF patient and his father. This variant was not detected in the other family members or the 600 Chinese Han control cases (Figure 1A and 1B), and were not found in the database of Human Gene Mutation Data base (HGMD), Ensembl and HapMap. Other atrial fibrillation–associated genes encoding ion channels and their accessory subunits were sequenced in the proband patient. However, no variant was detected in the proband patient.

**Table 1:** Clinical characteristics of the lone AF population (n=180)

Median age of onset, y (IQR)	34.2(26–41)
Male gender, %	72
Height, cm	171±6
Weight, kg	69±17
BMI, kg/m2	23.6±4.3
Blood Pressure, mmHg	
Systolic	127±16
Diastolic	76±10
AF type	
Paroxysmal, %	53.7
Persistent, %	31.7
Permanent, %	8.2
Unknown, %	6.4
Family history of AF	
First-degree relatives with AF (%)	32

All numbers are reported as mean±standard deviation unless otherwise noted. IQR Interquartile range.

The proband was a 38-year-old male patient affected with paroxysmal AF at the age of 28 years (ECG data: HR 102 bpm; no P-wave; QRS 98 ms) (Figure 1E). The patient had normal sinus rhythm ECG (HR 90 bpm; P-wave duration 98 ms; PR 168 ms; QRS 96 ms; QTc 392 ms) (Figure 1F) and his echocardiography was normal (left ventricular ejection fraction 57%; left atrium <40 mm). His father (age 66) was affected by persistent AF at age of 38 years and had the same mutation. Other family members were normal.

### Electrophysiological characterization of AF-associated T361S-K_v_4.3 mutant

**Figure 2:**
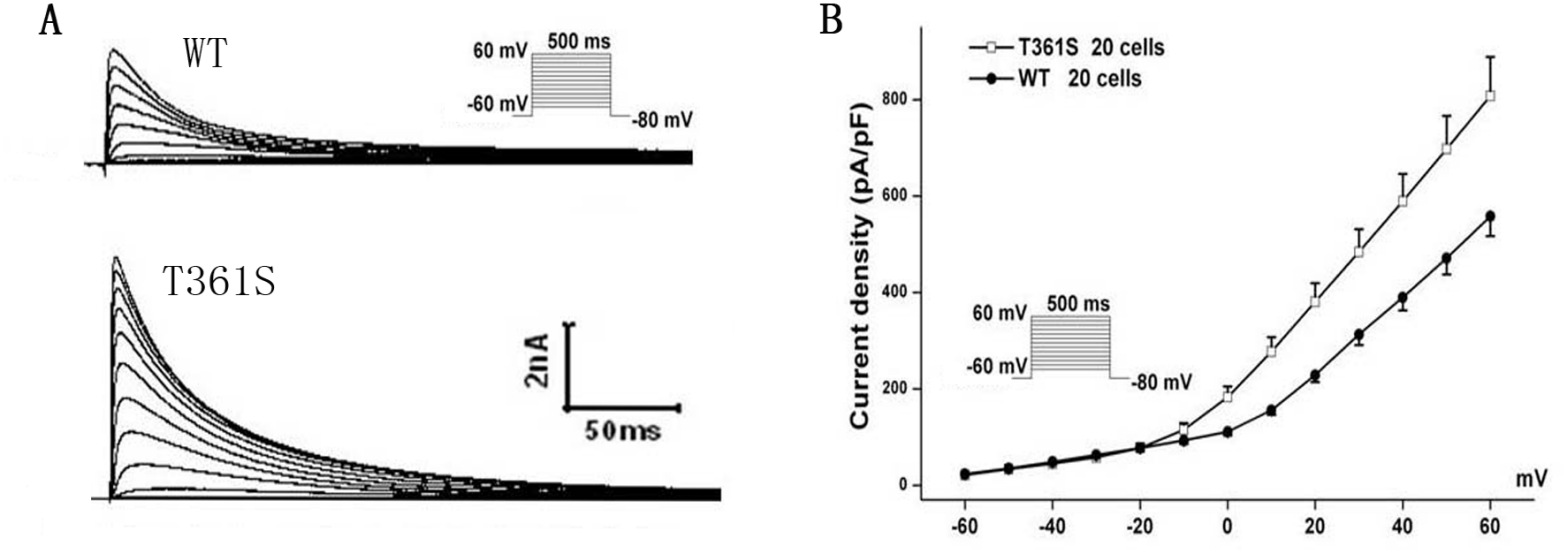
The mutant T361S-KCND3 prominently increases *I*_to_ density **(A)** Typical currents recorded from HEK293 cells transfected WT or T361S of K_v_4.3 together with KChIP2 in response to increasing step potential. **(B)** Current-voltage relationship. The protocol was shown in the inset. Data was shown as means ± SEM.

**Figure 3:**
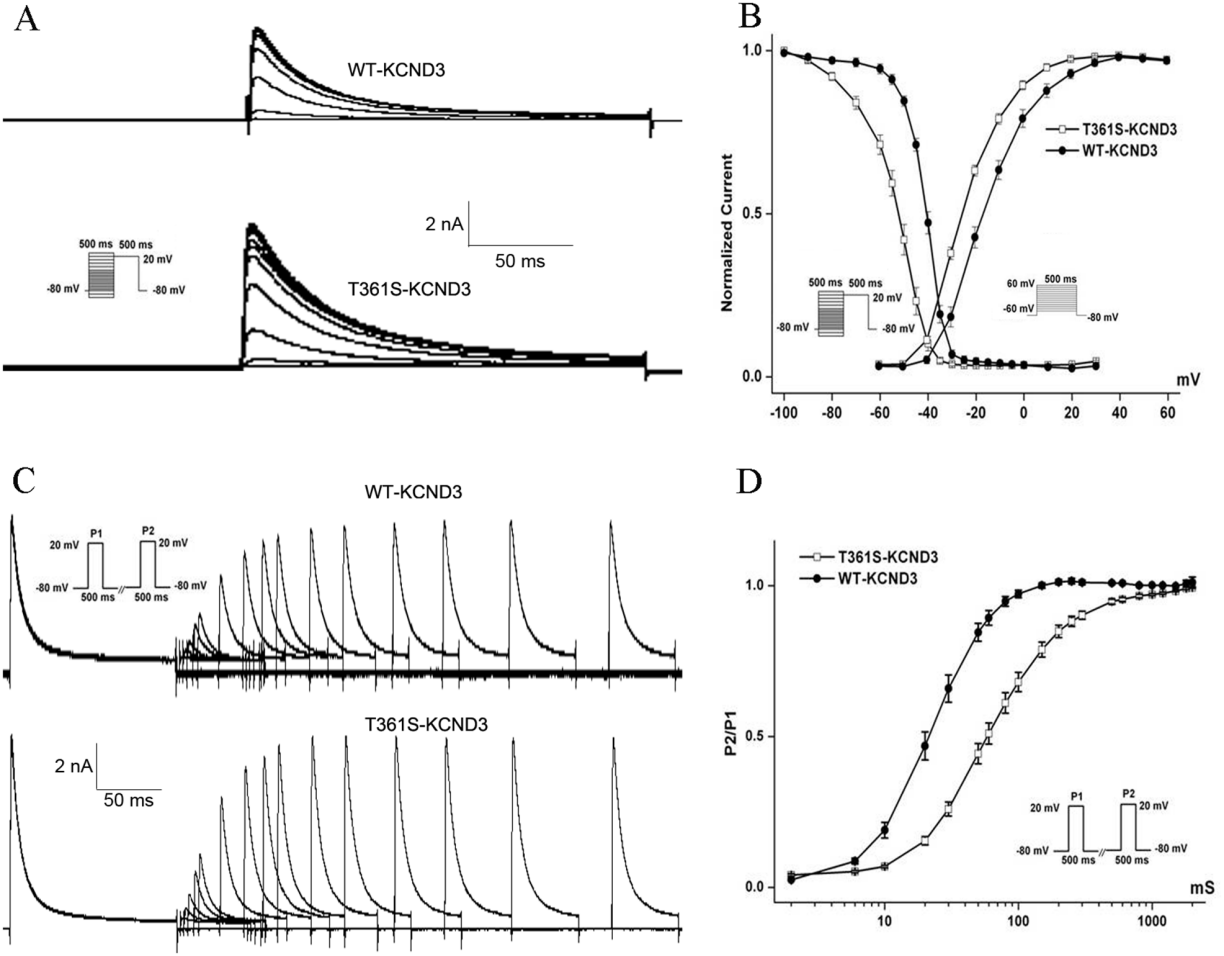
The mutant T361S-KCND3 alters the kinetic properties of *I*_to_ channel **(A).** Typical currents of voltage-dependent inactivation are shown. **(B)** Voltage-dependent inactivation and activation curves are plotted with Boltzmann equation. The recorded protocol was shown in the inset. **(C)** The curve of recovery from inactivation for WT and T361S channels is obtained using double pulse protocol. Representative recordings are shown. **(D)** The curve of recovery from inactivation. Peak *I*_to_ currents elicited by P2 were normalized (P2/P1) and the data is fitted with a single exponential function (X axis: time, ms; Y axis: P2/P1). Data was shown as means ± SEM.

The residue at the mutation position is highly conserved from Homo. sapiens to Xenopus. tropicalis (Figure 1D), suggesting that it is critical for the physiological function of the K_v_4.3 protein. Despites K_v_4.3 subunit generates K_v_ currents with similar to myocardial *I*_to_, additional subunits such as the regulatoryβcurrents and KChIPs are indispensable for the generation of cardiac *I*_to_ [24]. Thus, we recorded *I*_to_ under whole-cell voltage-clamp by transiently expressing WT or T361S-KCND3 together with KChIP2 in HEK293 cells. As shown in Figure 2A, cells expressing T361S-KCND3 had markedly increased *I*_to_ density (peak current normalized to cell capacitance, pA/pF) across the range of test potentials compared to WT-KCND3. The mutant T361S-KCND3 had an especially prominent enlargement of *I*_to_ density (807.79±80.99 pA/pF) at 60 mV compared to WT-KCND3 (557.9±40.61 pA/pF, *P*<0.01) (Figure 2B) by about 45%. Given that the mutation at position 361 in K_v_4.3 is located in the third extracellular loop linking S5 and S6, which is regarded as channel pore-forming (Figure 1C), it was necessary to analyze kinetic characteristics. As illustrated in Figure 3B, a significant hyperpolarizing shift of the steady-state activation curve was observed in the T361S mutant channel [WT: V_1/2_=-13.69±1.48, *k*=11.35±0.77, n=15 and T361S: V_1/2_=-22.98±0.75 (*P*<0.05), *k*=9.67±0.39, n=17], indicating a significantly negative shift of the activation curve of the T361S-KCND3 by 9.29 mV. A 13.14 mV negative shift of the steady state inactivation was also observed for the T361S channel (WT: V_1/2_=-41.16±0.47, k=5.37±0.39, n=17 and T361S: V_1/2_=-54.30±1.25 (*P*<0.05), k=8.56±0.36, n=18) (Figure 3B). Additionally, K_v_4.3 T361S expressed in HEK293 cells displays a slower recovery from the time-dependence of inactivation compared to the WT channel (T361S: t=81.17±10.18 ms; WT: t=27.33±2.80 ms; *P*<0.05, Figure 3C-3D). These results indicate that the T361S mutation results in a gain of function of *I*_to_.

### T361S mutants increased protein expression level in cell-surface

**Figure 4:**
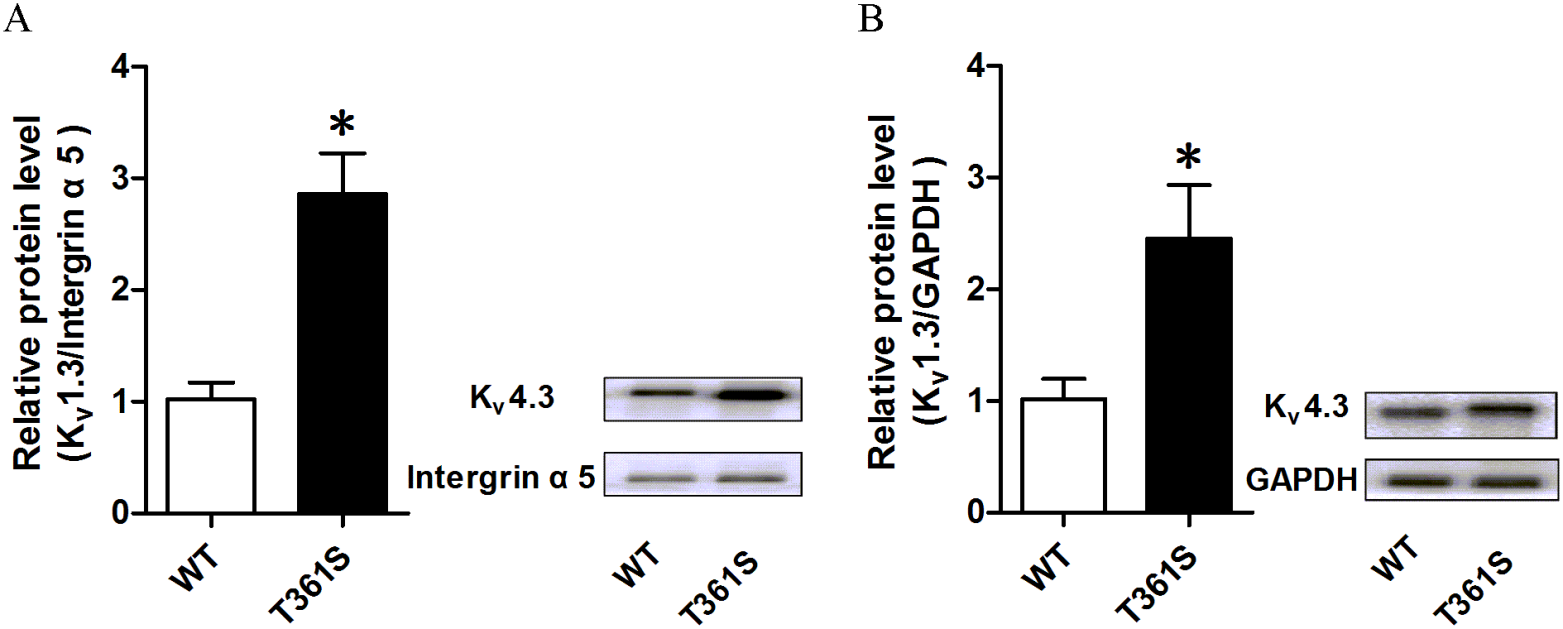
The effect of the missense mutation of K_v_4.3 on the cell-surface expression and the total expression of K_v_4.3 protein **(A)** Cell surface biotinylation assays were performed in Hek293 cells co-transfected KChIP2 with wild type or mutant T361S of K_v_4.3 after 48 hours. Intergrin α5 was used as loading to calibrate the cell-surface proteins. No GAPDH was detected in the biotinylated fraction (data not shown). **(B)** The total expression of K_v_4.3 protein was extracted from HEK293 cells co-transfected KChIP2 with wild type or mutant T361S of K_v_4.3. This experiment was repeated at least three times, and similar results were obtained. Data was shown as means ± SEM. ^*^P < 0.05.

The current density of K_v_4.3 is mainly determined by its expression level on the cell surface, the single channel conductance and the open probability of K_v_4.3 channel. To investigate the underlying molecular mechanism, the cell surface protein levels of the K_v_4.3 subunit were measured by western blot analysis. HEK293 cells were co-transfected KChIP2 with wild type or mutant K_v_4.3. As shown in Figure 4A, the T361S mutation up-regulated the expression of K_v_4.3 on the cell surface compared to wild type together with KChIP2 by about 2.8 folds. Interestingly, we also found that the expression level of mutant T361S in total cell extracts dramatically increased compared to WT (Figure 4B). Therefore, the results indicate that the up-regulation of the K_v_4.3 subunit in cellular surface contribute to the enlargement of *I*_to_ in T361S mutant.

### T361S shortened action potential duration through an increased *I*_to_

**Figure 5:**
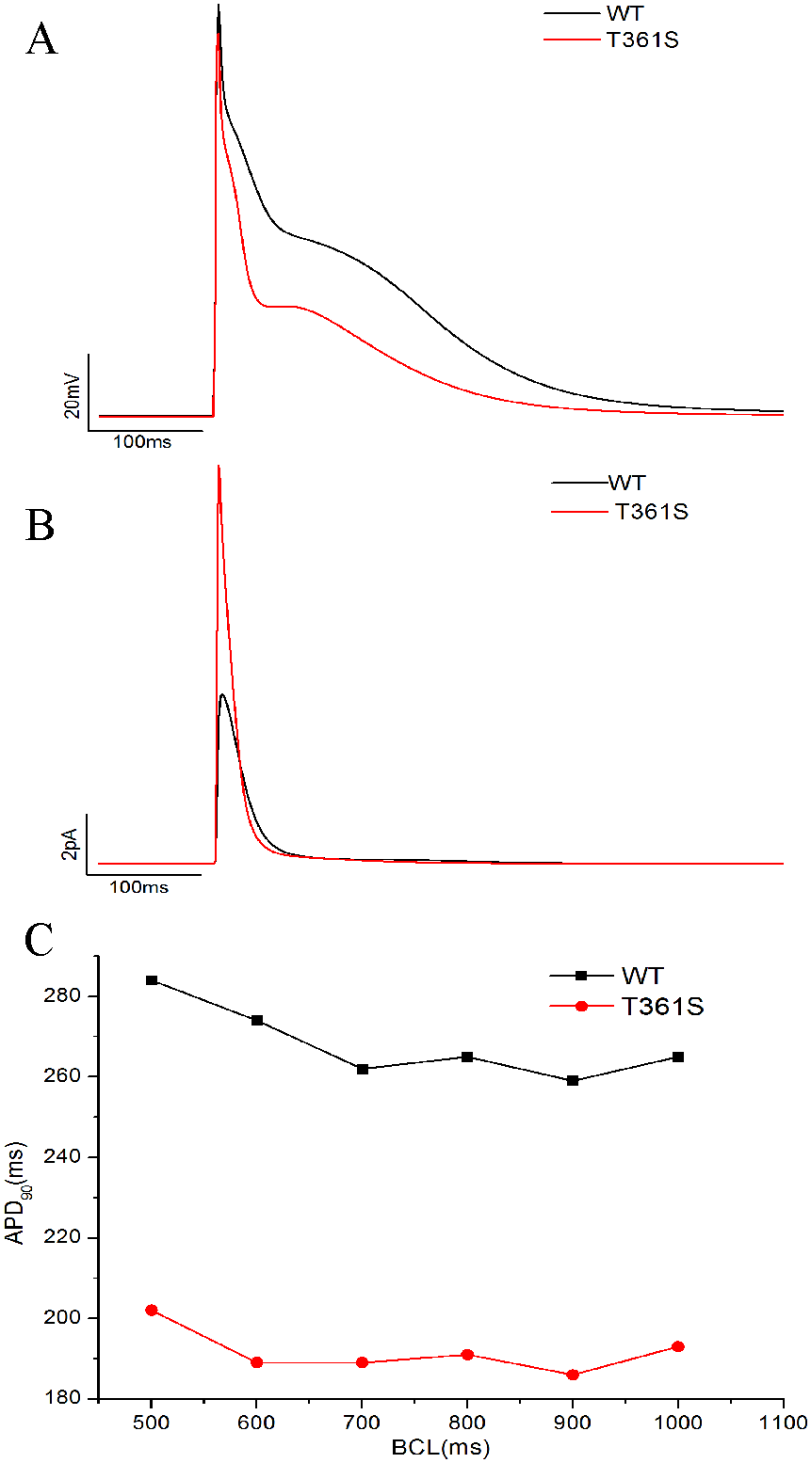
Computer simulation of the effect of the T361S mutation **(A)** Action potentials (APs) simulated at a BCL of 1000 ms. **(B)** The *I*_to_ current associated with the APs in (A). **(C)** Rate adaptation based on the model simulations. APD90 was calculated and plotted against BCL.

Compared to WT, the increase in the surface expression of the T361S mutation is by about 2.8 folds while the peak current at 60 mV only increased by about 145%, it suggest that per molecule activity of the T361S mutant channel is much lower than of WT (Figure 2B, Figure 4A). However, though computer simulation, the effect of the T361S mutation (in the presence of KChIP2), in the model, the mutation results in a shortening of the action potential duration through an increased *I*_to_ (Figure 5).

## DISCUSSION

In the present study, we carried out a sequencing analysis of the *KCND3* gene encoding the pore-forming K^+^ channel subunit K_v_4.3 in 180 AF patients. A novel mutation, T361S, was identified in a young patient with lone AF. The missense mutation resulted in a substitution of a threonine residue at position 361 in K_v_4.3 protein by a serine residue. The T361S mutation was absent in 600 control individuals and in publicly available databases. Electrophysiological studies revealed that the T361S mutation resulted in a gain of function compared to WT. In addition, the mutation facilitated the expression of K_v_4.3 protein subunits on the cellular surface and resulted in a shortening of the atrial action potential in Human Atrial Model. Collectively, our findings identify a novel T361S mutation associated with lone AF and suggest that a gain of function resulting from K_v_4.3 mutation may drive the pathogenesis of AF.

*I*_to_ channels are expressed in cardiomyocytes of larger mammals, and are important for early phase of repolarization and excitation-contraction coupling. Dysfunction of *I*_to_ channels in inherited or acquired cardiac diseases contributed to cardiac arrhythmias, including BrS and AF. In chronic AF patients, *I_t_*_o_ has been reported to be reduced by down-regulation of the K_v_4.3 mRNA levels [25, 26]. The efficacy of *I*_to_ blockers in terminating AF and a silico modeling suggested that reduced *I*_to_ may be a protective compensation during human chronic AF [27]. Olesen et al. first identified a gain of function mutation (A545P) in *KCND3* associated with AF in Danish populations, which led to reduce action potential duration in an atrial cell model, but the mechanism underlying the enlargement of the current amplitude by the A545P mutation remains unclear [23]. Recently, a genome-wide association study (GWAS) has identified a SNP (rs12044963) in *KCND3* gene, is relevant to AF susceptibility in the Japanese population [28]. Here, we observed T361S mutant dramatically increased *I*_to_ amplitudes, and up-regulated the surface and total K_v_4.3 expression in heterologous expression systems. Previous studies revealed that the translation efficiency in the threonine residue mutant (tcc) increased compared to the wild serine residue (acc), and a recent report has revealed that cold-inducible RNA-binding protein (CIRP) modulates cardiac repolarization by negatively regulating the expression of the K_v_4.2/K_v_4.3 proteins at the post-transcriptional level [29, 30]. We speculate that the mutation may disrupt the interaction of CIRP protein with *KCND3* mRNAs, increasing the translation efficiency which leads to an upregulation of protein levels of K_v_4.3 subunits. Enhanced *I*_to_ can accelerate atrial repolarization and shorten the atrial effective refractory period, which may facilitate reentry leading to the occurrence and maintenance of AF. Therefore, our findings expand the spectrum of mutations in *KCND3* that cause the pathogenesis of AF and various forms of cardiac arrhythmias.

Several mutations in *KCND3* were identified in dominantly inherited spinocerebellar ataxia (SCA) 19/22, early-onset of persistent lone atrial fibrillation, Brugada syndrome (BrS) and a case of sudden unexplained death [20, 22, 23, 31]. Mutant residues at positions 450 and 600 (L450F and G600R) associated with BrS significantly resulted in a gain of function and increased peak current density compared to WT [21, 32]. Because the ECG changes associated with BrS can be dynamic and are often concealed, and 10% to 20% of patients with BrS have AF, it is necessary to perform drug challenge to eliminate the BrS in the proband [33]. As ajmaline are unobtainable in China, propafenone challenge is also effective to reveal BrS as previously described [34]. The proband exhibited a negative reaction following propafenone challenge (data no shown). Interestingly, the mutant residues at positions 352 and 373 (T352P and M373I) in *KCND3* associated with SCA19 were also located in the third extracellular loop linking S5 and S6 [20]. However, the peak current density for the mutants was significantly reduced when compared to WT-KCND3. The results of the functional experiments were contrary to those for mutant residues at position 361 in *KCND3* associated with AF. Mutant residues at positions 352 and 373 (T352P and M373I) markedly reduced cell surface expression and enhanced protein degradation [20]. These results suggested that residues located in the extracellular loop linking S5 and S6 are crucial for the integrity of function of K_v_4.3 channel, and its mutation played an important and diverse role in the inherited diseases. This difference may be associated with tissue-specific gene expression, which remains to be investigated in the future.

As we used the candidate gene sequencing approach, other disease-associated genes cannot be ruled out. We limited our analysis to the *KCND3* encoding regions and the possibility of mutations occurring in regions beyond encoding regions cannot be excluded. However, lone AF is usually considered as a monogenic disease. A previous report revealed that gain-of-function mutations in *KCND3* are associated with AF [23], and our functional analyses point towards the identified *KCND3* mutation T361S as underlying AF.

Taken together, we identified a novel T361S mutation in *KCND3* associated with lone AF from a Chinese Han family, which results in a gain of function of *I_to_*. Mechanistically, the gain of function of *I_to_* elicited by the T361S mutation was associated with the increased expression of cell surface protein and total cell protein of K_v_4.3, which may be a molecular substrate leading to lone AF observed in our patient.

## MATERIALS AND METHODS

### Clinical data

This study was approved by the Ethics Committee of the First Affiliated Hospital of Xiamen University. Informed consent was obtained from all patients. The patients were enrolled through GeneID, a Chinese population database [35]. Patients with early onset lone AF (i.e., without other cardiovascular diseases, essential hypertension, ischemic stroke, metabolic or pulmonary diseases, or diabetes) and onset of AF before the age of 40 years were recruited. All the participants were diagnosed as lone AF based on the ACC/AHA/ESC 2006 AF guidelines by expert cardiologists using electrocardiogram (ECG) recordings. The proband exhibited a negative reaction following propafenone challenge. The 600 Chinese Han controls were normal healthy individuals without AF or any other cardiovascular diseases.

### Mutation analysis

Genomic DNA was extracted from peripheral blood samples using the Wizard Genomic DNA Purification Kit (Promega, Madison, Wisconsin, USA) according to the standard protocols. All of the exons and exon-intron boundaries of the candidate ion gene *KCND3* were amplified using genomic DNA from AF and the control patients as templates by polymerase chain reaction (PCR). The PCR products for the DNA sequencing were performed by Invitrogen Company (Shanghai, China) following the standard protocols. A variant was identified as a mutation if not present in the control population and NCBI database, associated with affected family members for segregation and affected a change of conserved amino acid.

### Construction of plasmids for KChIP2 and KCND3

T vectors containing cDNA encoding human *KChIP2* (NM_173192.2) or human *KCND3* (NM_172198.2) were purchased from Proteintech Company (Wuhan, China). The *KChIP2* cDNA was amplified by polymerase chain reactions (PCR) using PCR primers containing restriction enzyme sites (forward primer: 5-ATATCTCGAGATGCGGGGCCAGGGCCGCAAGGA-3 and reverse primer: 5-GCGCGGATCCTCTCCTGGGGGCTAGATGACAT-3), digested with the restriction enzymes Xho I and BamH I, and inserted into the vector pIRES2-EGFP. The *KCND3* cDNA was also amplified by PCR using PCR primers (forward primer: 5- ATATCTCGAGATGGCGGCCGGAGTTGCGGCCT-3 and reverse primer: 5-GCGCGAATTCTTACAAGGCGGAGACCTTGACA-3), digested with the restriction enzymes Xho I and EcoR I, and inserted into the vector pcDNA3.1 (-). The point mutation T361S (acc→tcc) in pcDNA3.1(-)-KCND3 was introduced using a PCR-based mutagenesis method (pcDNA3.1(-)-KCND3-T361S) as previously described [36, 37]. All of the constructed plasmids were verified by direct DNA sequencing analysis.

### Cell culture and transfection

HEK293 cells were cultured in Dulbecco’s Modified Eagle’s medium (DMEM) supplemented with 10% fetal bovine serum, L-glutamine (2 mM), penicillin G (100 μnits/ml) and streptomycin (10 mg/ml) in a humidified incubator with 5% CO_2_ at 37°C. The cells were transiently co-transfected with pIRES2-EGFP-KChIP2 and pcDNA3.1(-)-KCND3 (WT or T361S) at 70-80% confluence using GenJet™ *In Vitro* DNA Transfection Reagent (Signagen, Rockville, Maryland, USA) according to the manufacturer’s instructions.

### Electrophysiology

For patch clamp experiments, HEK293 cells in 24-well plates were transiently co-transfected with 0.5 μg of pIRES2-EGFP-KChIP2 and 0.5 μg of pcDNA3.1 (-)-KCND3 (WT or T361S). The experiments were performed 48 hours after transfection. A similar expression level of GFP-positive cells, which successfully expressed KChIP2 proteins, was selected to record *I*_to_. Cells were bathed in extracellular solution containing (in mM) 150 NaCl, 5 KCl, 2.2 CaCl_2_, 1 MgCl_2_, 10 HEPES, 5 glucose, pH 7.3 adjusted with HCl. Patch pipettes were filled with an internal pipette solution containing 120 K-gluconate, 24 KCl, 0.2 EGTA, 10 HEPES, pH 7.2 adjusted with KOH [18]. All reagents were purchased from the Sigma-Aldrich Company (ST.Lous, Missouri, USA). The currents were recorded at room temperature (22°C) using the whole-cell recording configuration and performed with an Axon MultiClamp 700B amplifier (Axon Instruments, Molecular Devices, Sunnyvale, California, USA). The pipette resistances ranged from 2-3 MΏ and the series resistances recorded in the whole cell configuration were compensated (85%) to minimize voltage errors. The holding potential for all pulse protocols was −80 mV, and the currents were low-pass filtered at 5 kHz with a 4-pole Bessel filter, digitized with the Digidata 1440A (Axon Instruments, Molecular Devices, Sunnyvale, California, USA) and sampled at 10 kHz. The data were acquired and analyzed using a software combination of Clamfit 10.2, Microsoft Excel, and Origin 8.5 (OriginLab, USA). The *I*_to_ densities (pA/pF) were obtained by dividing the peak current by the cell capacitance. The activation-potential and steady-state inactivation curves were all fitted with a Boltzmann equation (y= {1+exp [(V-V_1/2_)/k)]}^−1^) to determine the membrane potential for the half-maximal (in) activation V_1/2_, and the slope factor k. Recovery from inactivation was analyzed by fitting the data with a mono-exponential equation, y = y_0_+ [A_1_ exp(−x/τ_1_)], to determine the time constant τ1 for recovery from inactivation.

### Total cell protein and cell surface protein of K_v_4.3 for western blot analysis

Total protein lysates and plasma membrane proteins were extracted from HEK293 cells co-transfected KChIP2 with wild type or mutant K_v_4.3 after 48 hours following a standard protocol as previously described [38]. Plasma membrane proteins were isolated by the Pierce Cell Surface protein Isolation Kit (Thermo Scientific, Waltham, MA, USA). The total proteins or plasma membrane proteins were subjected to western blot analysis using a polyclonal anti-K_v_4.3 antibody (Alomone, Jerusalem, Israel) or GAPDH antibody (Proteintech Company, Wuhan, China).

### Computer simulation

Computer simulations were performed using the Grandi et al Human Atrial Model (HAR) [39]. The model code was obtained from the Grandilab (http://elegrandi.wixsite.com/grandilab/atrial-cell-models). All simulations were performed in Matlab (MathWorks, Natick, Massachusetts, USA) using the ode15s solver. To simulate the effects of the T361S mutation, the maximum conductance (g_to_) and current density for the *I*_to_ component of the HAR model was modified as follows: g_to_ was increased by a factor of 1.5 and current density was decreased by a certain factor. Based on the experimental data from measurements of WT+KChIP2 and T361S+ KChIP2 channels, the steady-state inactivation, steady-state activation curve, and I-V curve in the Grandi model are re-fitted. The simulations were performed at basic cycle lengths (BCLs) of 1000, 900, 800, 700, 600, and 500 ms. At each BCL, the action potential duration at 90% repolarization (APD_90_) was calculated for the 40th beat.

### Statistical analysis

All data were obtained from three independent experiments and expressed as the mean±SEM. The statistical analyses were carried out using a two tailed paired or unpaired Student’s t-test between two groups. The differences between groups over a time period were analyzed by two-way ANOVA. *P*<0.05 was considered statistically significant.
